# Using AI to Write a Review Article Examining the Role of the Nervous System on Skeletal Homeostasis and Fracture Healing

**DOI:** 10.1007/s11914-023-00854-y

**Published:** 2024-01-13

**Authors:** Murad K. Nazzal, Ashlyn J. Morris, Reginald S. Parker, Fletcher A. White, Roman M. Natoli, Jill C. Fehrenbacher, Melissa A. Kacena

**Affiliations:** 1 Department of Orthopaedic Surgery, Indiana University School of Medicine, Indianapolis, IN USA; 2 Department of Anesthesia, Indiana University School of Medicine, Indianapolis, IN USA; 3 Indiana Center for Musculoskeletal Health, Indiana University School of Medicine, Indianapolis, IN USA; 4 Stark Neuroscience Research Institute, Indiana University School of Medicine, Indianapolis, IN USA; 5 Richard L. Roudebush VA Medical Center, Indianapolis, IN USA; 6 Department of Pharmacology and Toxicology, Indiana University School of Medicine, Indianapolis, IN USA

**Keywords:** Artificial intelligence, AI, ChatGPT, Scientific writing, Fracture healing, Neural regulation

## Abstract

**Purpose of Review:**

Three review articles have been written that discuss the roles of the central and peripheral nervous systems in fracture healing. While content among the articles is overlapping, there is a key difference between them: the use of artificial intelligence (AI). In one paper, the first draft was written solely by humans. In the second paper, the first draft was written solely by AI using ChatGPT 4.0 (AI-only or AIO). In the third paper, the first draft was written using ChatGPT 4.0 but the literature references were supplied from the human-written paper (AI-assisted or AIA). This project was done to evaluate the capacity of AI to conduct scientific writing. Importantly, all manuscripts were fact checked and extensively edited by all co-authors rendering the final manuscript drafts significantly different from the first drafts.

**Recent Findings:**

Unsurprisingly, the use of AI decreased the time spent to write a review. The two AI-written reviews took less time to write than the human-written paper; however, the changes and editing required in all three manuscripts were extensive. The human-written paper was edited the most. On the other hand, the AI-only paper was the most inaccurate with inappropriate reference usage and the AI-assisted paper had the greatest incidence of plagiarism.

**Summary:**

These findings show that each style of writing presents its own unique set of challenges and advantages. While AI can theoretically write scientific reviews, from these findings, the extent of editing done subsequently, the inaccuracy of the claims it makes, and the plagiarism by AI are all factors to be considered and a primary reason why it may be several years into the future before AI can present itself as a viable alternative for traditional scientific writing.

**Supplementary Information:**

The online version contains supplementary material available at 10.1007/s11914-023-00854-y.

## Introduction

Artificial intelligence (AI) has been growing rapidly and has garnered significant attention in recent months [1]. Its capabilities and applications are numerous, and it can perform tasks in seconds that would take most human users significantly more time and effort. For this reason, AI has begun to inch its way into many different fields such as medicine and research [2, 3]. There have been many discussions in the research community on the use of AI in scientific writing. Some agree that AI can be a useful aid in writing, but many are against its use to write papers single-handedly or be listed as the author of a paper [4–7]. However, it is undeniable that AI can assist in scholarly writing. If AI can write believable abstracts [8], is it currently capable of writing a publishable full-length scientific review? To address this question, we created three experimental groups, with three papers per group. The first group wrote a review article without any use or input from AI (human). The second group, AI-only (AIO), used AI to do a literature review and in the writing of the manuscript. The third group, AI-assisted (AIA), used the same references and outline as the human group, but used AI in all text generation. There were three topics of review chosen and given to the separate authors. The topics for the papers were “Neural Regulation of Fracture Healing” [9–11], “COVID-19 and Musculoskeletal Health” [12–14], and “The Intersection of Alzheimer’s Disease and Bone” [15–18]. The final products were to be human, AIO, and AIA review articles written about each topic for a total of nine papers. Due to the AI’s information database ending in September 2021, the AIO paper of the COVID-19 was not able to be written, leaving eight papers. We hypothesized that the human paper would take the most time and require the fewest changes between the first and final drafts. We also hypothesized that the AIO paper would require the most changes but would take the least amount of time. Finally, we hypothesized that the AIA would take an intermediate amount of time and require fewer changes than the AIO paper. These hypotheses were made based on the assumption that AI can write text and review literature faster than humans but would have a higher rate of error. Refer to the *Introductory Comment* [19] of this edition for more information as well as specifics regarding the study design.

## Results

The amount of time that was spent on various activities for each of the three “Neural Regulation of Fracture Healing” review articles to make them of publishable quality is summarized in Table 1. Both the AIO and AIA manuscripts took less time to complete, 118.24 h and 164.63 h, respectively, when compared with the human manuscript, which took 167.94 h.

**Table 1:** The amount of time, in hours, that was spent on various activities for each of the three “Neural Regulation of Fracture Healing” review articles

Activity	Human	AI-only	AI-assisted
Preparation (h)	0	5.32	8.33
Literature review (h)	42.63	0	42.63
Outline (h)	7.57	3.22	7.57
Writing (h)	32.09	10.81	32.94
AI fact checking (h)	0	27.12	7.43
Student edits (h)	65.90	52.03	40.66
Faculty edits (h)	18.50	14.12	22.23
Other (h)	1.25	5.62	2.84
Total time (h)	167.94	118.24	164.63

The level of similarity between the original and final drafts of each manuscript is shown in Table 2. Please see Supplementary Materials 1–3 for the original drafts of the human, AIO, and AIA manuscripts, respectively. The original drafts used for this comparison were completely generated by ChatGPT for both the AIO and AIA manuscripts. Each final manuscript was given a similarity score to its original draft based on the sum of the percent of its sentences that were identical to those in the first draft, had been altered by only minor changes, or had been paraphrased. The human manuscript had a similarity score of 47.4%, the AIO manuscript had a similarity score of 56.5%, and the AIA manuscript had a similarity score of 51.1%.

**Table 2:** The level of similarity between the original and final drafts of each of the three “Neural Regulation of Fracture Healing” review articles

Level of similarity	Human	AI-only	AI-assisted
Identical (%)	5.3	11.9	8.6
Minor changes (%)	10	22.6	15.1
Paraphrased (%)	32.1	22.0	27.4
Different (%)	52.6	43.5	48.9

Supplementary Materials 4 and 5 list all the queries required to generate the AIO and AIA review articles. It took 254 queries to generate the first draft of the AIO manuscript and 416 queries to generate the first draft of the AIA manuscript. Given significant limitations on the level of detail included in the original AIO manuscript since ChatGPT was not fed the entire text of each source cited, revisions to this paper were completed by the author and not by querying ChatGPT. Conversely, since ChatGPT had access to the complete text of each source used to write the AIA paper, and so wrote with an appropriate level of detail, this manuscript was edited by querying ChatGPT 232 additional times (total of 648 queries) before making a limited number of edits by hand.

The accuracy of the references used by ChatGPT in the first iteration of the reviews is shown in Table 3. ChatGPT included 55 references in its original draft of the AIO manuscript and 43 (78.2%) of these were removed to generate the final draft of the manuscript. Of those removed, 14.0% (6 references) were removed as the cited source did not exist and 86.0% (37 references) were removed as the content of the source did not match the content of the associated sentence. The author added 134 references in the process of editing the manuscript, and the final draft of the manuscript cited 146 references. Thus, the final manuscript was composed of 8.2% AI-generated sources (12 sources) and 91.8% human-generated citations (135 sources). In the AIA review, there were 176 in-text citations in the original draft. Of these, 13.6% were found to be plagiarizing content from another paper (24 citations). Overall, the AIA review had the highest plagiarism similarity index in its first draft, with an index of 39% (a level suggesting plagiarism is likely) which was intentionally edited to reduce it, resulting in a plagiarism similarity index of 16% in the final draft. While the 16% plagiarism similarity index is considered acceptable (anything under 24%), it is still much higher than that observed in the first and final drafts of the human and AIO manuscripts. These results are shown in Table 4.

**Table 3:** The number and accuracy of references generated in the first draft of the AIO and AIA fracture healing review articles

Reference criteria	AI-only	AI-assisted
AI-cited references	55	176
Number correctly cited	12	152
Number incorrectly cited	43	24
% incorrectly cited	78.2%	13.6%

**Table 4:** The plagiarism similarity indices of the first and final drafts for each of the three neural regulation of fracture healing review articles

Draft version plagiarism similarity index (%)	Human	AI-only	AI-assisted
First draft	2%	6%	39%
Final draft	3%	7%	16%

## Discussion

This project aimed to investigate the capabilities of AI in conducting scientific writing. The process of scientific writing is multifaceted and requires many different elements: time, a thorough review of relevant literature, ethical use of referenced literature, a comprehensive understanding of scientific writing, accuracy, and editing. Our primary objective was to identify how AI compares to humans in these metrics.

Regardless of the modality used, AI reduced the number of hours required to write a review by a wide margin. The human-written paper took over 35 h more to complete when compared to either AI paper. When examining the difference between the AIO and human paper, three key time differences became evident. The human paper required a literature review and took longer to physically write. AIO conducted no real literature review but rather pulled directly from its knowledge base to write immediately, and it was a faster writer. On the other hand, AIO required extensive fact-checking compared to the human paper. The AIA paper took a similar amount of time to write as the human paper, primarily due to the large number of queries required to complete the paper. The same literature review time was given to both the human and the AIA papers, as all references used in the AIA paper were found in the human-conducted literature review. However, the actuality of the AIA paper is that it would require significantly less time to conduct a literature review. The literature review conducted for the human paper encompassed both finding the appropriate sources and reading the articles. If the literature review was conducted solely for the AIA paper, it would only require identification of appropriate sources and brief skimming of the articles versus the full-length reading, thus saving time. Overall, the AIA and AIO papers took less time to write than the human-written review and have the potential to save time in scientific writing.

When examining other aspects of the writing process, it became clear that each review presented its own unique challenges and weaknesses. The primary issue found in the human-written paper, in addition to the time spent, was the extent of editing. This paper spent the most time in edits and had the lowest similarity score, primarily to rewrite sections to improve readability.

The AIO review presented two problems which were, in essence, the exact opposite of those found in the human paper. While the AIO paper had excellent writing technique, the content was both superficial and inaccurate. The superficiality and inaccuracy of the paper required extensive human editing, so it also had a low similarity score and spent a similar amount of time in the editing process. The inaccuracy bled into the references used, and because of this over 90% of the references in the AIO review were added manually during editing.

It may be assumed that human issues with writing flow and AI factual inaccuracies may be remedied by a human-conducted literature review and then using ChatGPT for writing purposes. This is mostly true, as the AIA paper had a similar ease of readability as the AIO paper and was just as accurate as the human paper. However, the AIA paper brought to light the challenge of plagiarism. To generate the AIA review, ChatGPT reads the referenced article and draws conclusions. During that process, it appeared to oftentimes describe the study by directly copying the abstract of that article. This issue undoubtedly had to be addressed, and a thorough examination of the AIA paper was conducted with subsequent editing to certify that the review would not be considered plagiarized. This was evaluated using the Turnitin software and examining the plagiarism similarity indices by comparing the manuscript to published literature and sources on the internet. Typically, a plagiarism similarity index of under 24% is “green” or not considered to be plagiarized. As such, although the first draft of the AIA paper was “yellow” and thus can be flagged for potential plagiarism, the final draft of the review was under the 24% threshold.

All review articles were peer-review as per standard *Current Osteoporosis Reports* procedure. During the revision stage, the AIO paper came back with only minor comments and changes. However, the human and AIA papers came back with more extensive revisions, both primarily focused on reorganization of the text. The critique similarities, primarily concerns about ineffective flow of ideas, may suggest that AI is able to generate outlines with more clarity and better organization than humans, revealing another important advantage of using AI.

The primary objective of this endeavor was to demonstrate the current capacity of AI to generate an appropriate scientific review. We have discovered that, with no human guidance, AI is not yet capable of writing an appropriate, publishable review article due to its fabrication of references, inaccuracies, and lack of detail. Nonetheless, it seems as though its writing ability from a non-scientific, standard essay standpoint is equivalent to or better than the average individual. Moreover, as observed in the AIA paper, it can potentially augment human writing and simultaneously reduce the time required to write, although manual examination of the written text is required to ensure ethical writing. While AI, as it currently stands, likely requires years of learning/updating before it can be seriously considered to write a scientific review with minimal human intervention, it can currently be used as a tool for improved human writing and efficiency.

### Supplementary Information

Below is the link to the electronic supplementary material.

Supplementary file1 (DOCX 816 KB)

## Data Availability

No datasets were generated or analysed during the current study.
